# Ultrasound-Guided Neuraxial Anaesthesia for Vaginal Hysterectomy in a Patient With Rheumatoid Arthritis

**DOI:** 10.7759/cureus.35064

**Published:** 2023-02-16

**Authors:** Amreesh Paul, Anjali Borkar

**Affiliations:** 1 Anaesthesiology, Jawaharlal Nehru Medical College, Datta Meghe Institute of Higher Education and Research, Wardha, IND

**Keywords:** difficult spinal anaesthesia, difficult epidural, rheumatoid arthritis, ultrasound guided spinal anaesthesia, ultrasound-guided, ultrasound-guided epidural

## Abstract

Rheumatoid arthritis is a type of chronic inflammatory arthritis affecting about 1% of the population. Females are more frequently affected than males. The aetiology of the disease is uncertain. Immunological, genetic and environmental factors play a role in the manifestations. The condition is characterised by a combination of articular symptoms and multi-organ involvement. These pose a wide array of difficulties in administering anaesthesia for patients with rheumatoid arthritis. These patients are prone to have a problem in airway management due to arthritic changes in the cervical spine and temporomandibular joint and in the administration of neuraxial anaesthesia owing to changes occurring in the vertebral column. We present a case of a 45-year-old female with rheumatoid arthritis posted for a vaginal hysterectomy. The patient had narrowed intervertebral spaces and was managed successfully by the use of ultrasonography to place an epidural catheter and for the administration of subarachnoid block for the conduct of the procedure.

## Introduction

The most prevalent inflammatory arthritis, affecting 1% of adults, is rheumatoid arthritis (RA). Females are two to three times more likely than males to develop RA. Although the cause is uncertain, it is thought to result from a complex interaction between immunological, genetic, and environmental factors [1]. Exacerbations and remissions define the course, and the manifestation might be acute or chronic. Symmetric polyarthropathy and systemic involvement are typical characteristics. Both articular and extra-articular lesions primarily affect the small joints [2]. Furthermore, non-articular muscle tissues such as tendons, ligaments, and fascia are susceptible to RA. About 40% of people with RA experience extra-articular symptoms at some point early on or later on in the disease. Rheumatoid factor and HLA-DR4-positive individuals are more prone to experience extra-articular involvement. Its defining features are destructive polyarthritis and extra-articular organ involvement, such as skin, eyes, hearts, lungs, kidneys, neurological, and gastrointestinal systems [3]. 

Anaesthetic management of patients with RA can be challenging due to the arthritis of various joints and systemic involvement. Cervical spine involvement is seen very often. Atlanto-axial subluxation and consequent separation of the Atlanta-odontoid articulation are mainly seen in the cervical spine [1]. The temporomandibular joint may also be involved and associated with pain during mastication or asymptomatic. These factors may make airway management difficult in patients with RA [4]. There is also the risk of rapid desaturation during apneic laryngoscopy despite adequate pre-oxygenation and denitrogenation due to restrictive lung disease that may be seen with RA.

Regional anaesthesia should be considered whenever possible, as it avoids airway manipulations and may provide good post-operative pain relief. Regional block administration, however, could be technically challenging due to severe lumbar and thoracic spine arthritis. Anatomical landmarks may be distorted due to contractures or deformities [5]. Synovitis and degenerative changes in the facet joints of the lumbar spine cause pain and instability. Endplate erosions are present in RA lumbar lesions. Due to inflammatory degeneration of the collagen between the disc and endplates, there is a decrease in disc space and instability. Additional symptoms include erosive discitis, spinal stenosis, vertebral collapse, and an amplification of the inflammatory response from the apophyseal joints. In about 28% of patients, the combination of these modifications causes lordosis and degenerative scoliosis loss [6].

We present a case of a 45-year-old female with RA-associated skeletal complications posted for vaginal hysterectomy and the anaesthetic management of the case.

## Case presentation

A 45-year-old female presented with complaints of abdominal pain and increased frequency of micturition for three months. The patient was asymptomatic until one year back when she noticed vaginal prolapse while squatting or lifting heavy weights. The abdominal pain was stabbing in nature and associated with nausea and white discharge. A bimanual pelvic examination revealed Grade II utero-cervical descent, cystocele, rectocele, and enterocoele. Ultrasound examination revealed a large uterus with multiple uterine fibroids, with the largest measuring 20*15*12 mm. The patient was planned to be posted for a vaginal hysterectomy.

Pre-anaesthetic assessment

Pre-anaesthetic checkup of the patient revealed that she was a known case of RA for around 35 years, not on regular medications. The BMI of the patient was 23.4 kg/m2. She reported a history of morning stiffness and stiffness of both the inter-phalangeal and wrist joints. There was no history of peripheral neuropathy, Raynaud's phenomenon, or ocular involvement. She had a swan neck deformity of the right fifth finger. She was on methotrexate 15mg twice a week, hydroxychloroquine sulphate 400mg/day, and deflazacort 12mg/day. The patient had no other co-morbidities, good exercise tolerance, and normal systemic examination. Mouth opening was adequate, with a Mallampati grade of III. Pulse rate was 78 bpm, blood pressure was 114/72 mmHg, R.R. was 17 per min, and SpO_2_ was 99% on room air. The patient had decreased neck mobility, and palpation of the lumbar spine revealed minimal intervertebral spaces. Complete blood count, renal function tests, liver function tests, chest x-ray, ECG, and ECHO were within normal limits. R.A.'s factor was 59.9 IU/mL. An x-ray of the cervical spine and lumbar spine was obtained.

**Figure 1:**
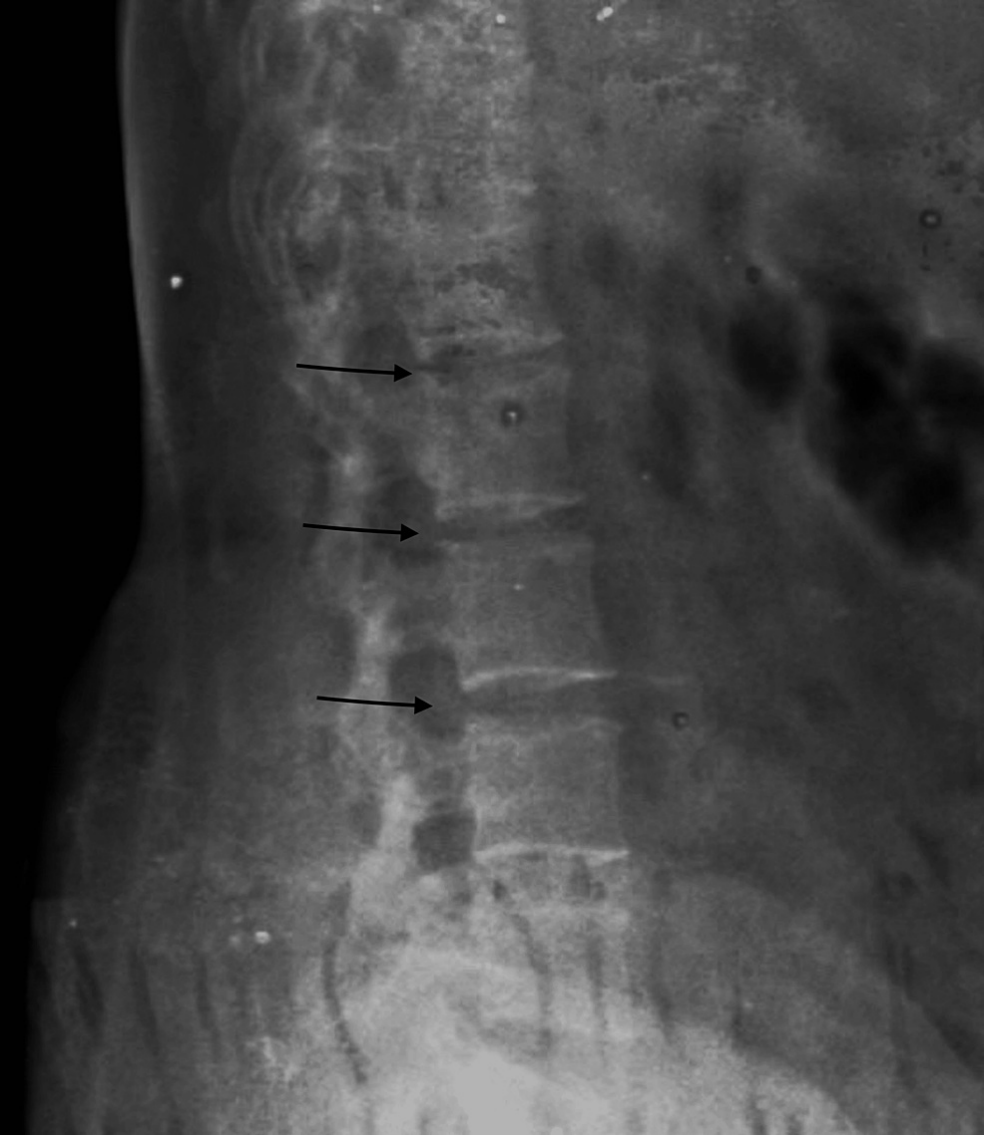
Lateral x-ray of the Lumbar Spine showing decreased intervertebral space

A cervical x-ray showed features of cervical spondylosis, and a lumbar x-ray revealed loss of lumbar curvature with decreased intervertebral spaces (Figure 1). Hence it was decided to employ ultrasound-guided epidural catheterisation and spinal anaesthesia as an anaesthetic modality for the surgery, with general anaesthesia as a backup. Written and informed consent was taken for the same after explaining the risk of failed neuraxial technique and anticipated difficult intubation.

Anaesthetic management

After confirming the nil per os duration, the patient was transferred to the operating room on the day of the procedure. After establishing a good peripheral venous access, intravenous fluid therapy was initiated. Monitoring equipment for the ECG, pulse oximetry, non-invasive blood pressure, and heart rate were attached. All the baseline parameters were observed and recorded (pulse rate -76/min, NIBP - 119/81mmHg, RR - 16/min, and SpO_2_ - 100% on room air). The patient was positioned in the left lateral position with hips and knees flexed.

The ultrasound scan was performed with a 3-13 MHz linear probe in the paramedian sagittal plane (1-2cm away from the spinous process, with the orientation marker directed cranially) to visualise the posterior epidural space at the level of L3-L4 interspace, anechoic CSF at the level of L4-L5 interspace and the sites were marked using a surgical pen. The parts were then painted and draped, following which an adequate amount of ultrasound gel was applied to the probe. The probe and the cord were wrapped in a sterile plastic sleeve. The probe was held in the operator's non-dominant hand and positioned at the site previously marked. With the two vertebrae in view, 2mL of lignocaine 2% was injected into the skin and subcutaneous tissue.

The 18G Tuohy's needle was inserted using an in-plane approach from the caudal end, advancing the tip gradually towards the ligamentum flavum (kept at the centre of the screen) by a second anaesthesiologist. Once the needle was in the ligamentum flavum, the needle was advanced very carefully while checking for loss of resistance in the LOR syringe. Loss of resistance was encountered once the needle was in the epidural space. A catheter was threaded through Tuohy's needle, and 3cm of it was introduced into the epidural space and fixed externally using a sterile dressing. Spinal anaesthesia was administered following the same technique with a 25G Quincke needle and 17mg of hyperbaric bupivacaine. The patient was immediately positioned supine. The sensory level of the blockade was assessed using the pinprick method along the mid-clavicular line. After a sensory blockade corresponding to the T6 dermatome was achieved, the procedure was commenced. The epidural catheter was injected with 3mL of lignocaine-adrenaline (1:200,000) to confirm placement in the epidural space. The procedure lasted two hours, and the vital parameters were stable. Post-procedure, the patient was monitored in the post-operative unit for four hours before being shifted to the ward. The patient was given 8mL of 0.125% of isobaric bupivacaine twice for two days in the postoperative period, after which it was removed. The patient was discharged from the hospital on POD 8.

## Discussion

RA is a chronic inflammatory arthritis. Symmetric poly arthropathy, extra-auricular lesions, and the involvement of multiple organ systems characterise it. A thorough pre-anaesthetic assessment should be carried out to rule out any possible systemic involvement and devise an appropriate anaesthesia administration strategy. The clinical manifestations of RA can make airway management and conduct of general anaesthesia challenging. Airway management in these patients would require very minimal movement of the neck during laryngoscopy. It is preferable to employ regional anaesthesia whenever possible. The deformities associated with the disease and its limitations on the patient's positioning must be considered when planning for regional anaesthesia. As seen with this patient, even neuraxial anaesthesia could have been difficult if not for the use of ultrasonography.

Epidural anaesthesia and analgesia are frequently used in the perioperative and postoperative periods in infra-umbilical and lower limb surgeries. It provides superior analgesia over intravenous analgesics [7]. The successful placement of the catheter in the epidural space by landmark-guided technique is highly dependent on the identification of lumbar interspaces [8]. In cases where identification is difficult, or the lumbar interspace is decreased, it is challenging to employ neuraxial anaesthesia. Hence, ultrasound guidance can be used in such cases to increase the success of the techniques and avoid the administration of general anaesthesia for these procedures. In this instance, there was decreased lumbar interspace and anticipated difficult intubation. The usage of ultrasonography made the implantation of neuraxial anaesthesia much more feasible for the patient. The real-time visualisation of the spinal structures helped place the catheter with relative ease and patient satisfaction. Incidences of accidental dural puncture with the epidural needle can be avoided as the needle is visualised throughout the procedure and due to the fact that the CSF is anechoic on ultrasonography. The main disadvantages associated with using ultrasonography are the increased costs and the need for two providers for epidural catheter placement and handling the ultrasound [9].

## Conclusions

RA is associated with multiple features that make anaesthetic management difficult. General anaesthesia and neuraxial anaesthesia can be difficult to administer owing to various reasons from anatomical distortions to multi-organ involvement. Using ultrasonography for neuraxial anaesthesia can make neuraxial anaesthesia feasible under such challenging situations. With the rapid advancement in technologies in the healthcare industry, the anaesthesiologists must be adept with them to provide safe and better care for patients.
